# Many Ways to Rome: Exercise, Cold Exposure and Diet—Do They All Affect BAT Activation and WAT Browning in the Same Manner?

**DOI:** 10.3390/ijms23094759

**Published:** 2022-04-26

**Authors:** Anna K. Scheel, Lena Espelage, Alexandra Chadt

**Affiliations:** 1 Institute for Clinical Biochemistry and Pathobiochemistry, German Diabetes Center (DDZ), Leibniz-Center for Diabetes Research at the Heinrich Heine University, Medical Faculty, Düsseldorf, Auf’m Hennekamp 65, 40225 Duesseldorf, Germany; anna.scheel@ddz.de (A.K.S.); lena.espelage@ddz.de (L.E.); 2 German Center for Diabetes Research (DZD), Partner Düsseldorf, München-Neuherberg, 85764 München, Germany

**Keywords:** insulin resistance, obesity, T2DM, exercise metabolism, BAT activation, WAT browning, adipokines and myokines

## Abstract

The discovery of functional brown adipose tissue (BAT) in adult humans and the possibility to recruit beige cells with high thermogenic potential within white adipose tissue (WAT) depots opened the field for new strategies to combat obesity and its associated comorbidities. Exercise training as well as cold exposure and dietary components are associated with the enhanced accumulation of metabolically-active beige adipocytes and BAT activation. Both activated beige and brown adipocytes increase their metabolic rate by utilizing lipids to generate heat via non-shivering thermogenesis, which is dependent on uncoupling protein 1 (UCP1) in the inner mitochondrial membrane. Non-shivering thermogenesis elevates energy expenditure and promotes a negative energy balance, which may ameliorate metabolic complications of obesity and Type 2 Diabetes Mellitus (T2DM) such as insulin resistance (IR) in skeletal muscle and adipose tissue. Despite the recent advances in pharmacological approaches to reduce obesity and IR by inducing non-shivering thermogenesis in BAT and WAT, the administered pharmacological compounds are often associated with unwanted side effects. Therefore, lifestyle interventions such as exercise, cold exposure, and/or specified dietary regimens present promising anchor points for future disease prevention and treatment of obesity and T2DM. The exact mechanisms where exercise, cold exposure, dietary interventions, and pharmacological treatments converge or rather diverge in their specific impact on BAT activation or WAT browning are difficult to determine. In the past, many reviews have demonstrated the mechanistic principles of exercise- and/or cold-induced BAT activation and WAT browning. In this review, we aim to summarize not only the current state of knowledge on the various mechanistic principles of diverse external stimuli on BAT activation and WAT browning, but also present their translational potential in future clinical applications.

## 1. Insulin Resistance—The Link between Obesity and Type 2 Diabetes Mellitus

Global rates of obesity and associated metabolic diseases are on a constant rise [1]. In addition to a familial genetic predisposition [2], abdominal obesity often emerges from caloric excess and simultaneous decline of energy expenditure [3,4] resulting in increased white adipose tissue (WAT) mass and adipocyte expansion [5]. Abdominal or visceral adiposity is closely linked to various metabolic impairments. One key feature of this form of adiposity is the so-called “adipocyte hypertrophy”, a compensation of this fat depot towards the increased demands in fat storage by enlargement of the pre-existing cells. In particular, large adipocytes with concomitantly reduced mitochondrial density have been associated with insulin resistance (IR), dyslipidemia, and increased risk of Type 2 Diabetes Mellitus (T2DM) [6,7,8]. Furthermore, abdominal or visceral obesity represents the predominant risk factor for the development of the so-called “metabolic syndrome” [4,9,10,11,12,13] which is defined as a co-occurrence of metabolic abnormalities, including abdominal obesity with concomitant dyslipidemia and hypertension, as well as glucose intolerance and IR [14,15]. IR represents a characteristic trait of T2DM and is defined by an inadequate response of insulin-responsive tissues, such as skeletal muscle and adipose tissue, to pancreatic insulin secretion [16]. The risk to develop IR is exacerbated by an inactive, sedentary lifestyle [17] in combination with overnutrition and successional obesity [13]. In contrast, regular exercise, which has been recommended as 150 min/week of moderate to vigorous physical activity [18], can reduce the susceptibility towards obesity and T2DM [19].

IR in skeletal muscle and adipose tissue is considered a major factor during T2DM development. Ectopic lipid accumulation and dyslipidemia in non-adipose tissues, especially skeletal muscle or the liver, have been identified as detrimental in the pathogenesis of obesity and the related lipid-mediated IR [20]. On a cellular level, IR is often caused by disturbances in the insulin signaling cascade. Harmful lipid metabolites such as ceramides interfere with insulin signal transduction [21] in a variety of individual steps. Insulin receptor signaling has been demonstrated to be decreased by fatty acids (FAs) such as palmitate [22]. Furthermore, the insulin receptor substrates 1 and 2 (IRS1/2) [23] present major targets for the induction of insulin resistance, for instance due to the accumulation of specific lipid metabolites [24]. In particular, diacylglycerols (DAGs) have been shown to stimulate protein kinase C (PKC) isoforms, a process that results in inhibitory serine phosphorylation of IRS1 and 2 [25,26].

Another example for lipid-induced IR is the activation of the Toll-like Receptor 4 (TLR4) by FAs [27], which mediates a pro-inflammatory inhibition of AKT phosphorylation. Moreover, lipid metabolites or triglycerides have been identified as circulating biomarkers to predict IR in the onset of T2DM [28,29,30]. Adipose tissue IR further contributes to this effect by increasing lipid availability due to elevated basal lipolysis and impaired anti-lipolytic action of insulin [21,25,31].

## 2. Skeletal Muscle and Adipose Tissue in Health and Disease—Metabolic Flexibility

In the healthy, metabolically flexible state, the organism displays a large potential to adapt to nutritional conditions on demand. During fasting periods, lipolysis in adipose tissue and FA oxidation (FAO) in skeletal muscle are upregulated to provide fuel for other organs. In the post-prandial, thus insulin-stimulated state, glucose uptake into skeletal muscle and fat tissue, as well as glycogen and lipid synthesis are accelerated. Metabolic flexibility also includes fuel adaptation in response to exercise to meet the high energy requirements during physical activity. IR has been implicated with an organism’s inability to adapt fuel oxidation to changes in fuel availability, a state that consequently has been described as “metabolic inflexible”. Many T2DM patients demonstrate a reduced ability to switch between lipid and carbohydrate utilization in response to the acute metabolic demand [6,7].

Whole-body glycaemia is regulated by a network of tightly interconnected organs, communicating in response to nutritional and physiological challenges [8]. The skeletal muscle represents the primary regulator of post-prandial glucose clearance by accounting for over 80% of glucose disposal [32]. Upon insulin stimulation, glucose enters skeletal muscle and adipose tissue via the specialized glucose transporter 4 (GLUT4). In the basal state, GLUT4 is mainly located in intracellular GLUT4-storage vesicles but translocates to the plasma membrane in response to insulin or (in skeletal muscle) to contraction [33,34]. Whereas the global knockout (KO) of GLUT4 in mice leads to impaired growth developmental and cardiac hypertrophy, muscle-specific KO of GLUT4 in mice (mG4KO) results in an insulin-resistant and mildly diabetic phenotype [35,36], highlighting the importance of skeletal muscle glucose disposal for whole-body glycaemia.

The adipose tissue holds an important regulatory function of energy balance and glucose metabolism as well. It can be classified into three major subtypes: (i) WAT, (ii) brown adipose tissue (BAT), and (iii) brown-like/beige or brite (brown in white) adipose tissue. The WAT is mainly located under the skin (subcutaneous (sc)WAT) and in the abdominal cavity between the organs (visceral (vis)WAT), and primarily specializes in storing excess lipids, mechanical cushioning, and promoting thermal protection [37]. Despite the fact that WAT only accounts for less than 10% of whole-body glucose uptake, adipose tissue-specific GLUT4 knockout (aG4KO) in mice leads to glucose intolerance and insulin resistance, with individual animals even developing severe IR and diabetes [36,38]. It has been demonstrated that aG4KO mice display a reduction of approximately 50% on in vivo glucose uptake. Interestingly, in vivo but not ex vivo glucose uptake into skeletal muscle is also impaired in these mice, indicating an important inter-organ crosstalk between these two tissue types [34]. Consequently, combined knockout of GLUT4 in adipose tissue and skeletal muscle in mice (amG4KO) leads to fasting hyperglycemia, glucose intolerance, and an elevated risk to develop IR compared to both aG4KO and mG4KO, which emphasizes the key role of the adipose tissue-skeletal muscle connectivity in glucose homeostasis [39]. The regulatory communication or “crosstalk” between these tissues is mediated via numerous circulating factors. Both the skeletal muscle and the adipose tissue have been described as extremely active in secreting various proteins in response to external stimuli. These factors have been termed “myokines” and “adipokines”, related to their respective organ of origin [31].

In contrast to the WAT, BAT is rich in mitochondria. The high mitochondrial density is in fact responsible for the brownish coloring of this tissue [40]. The activated BAT represents the predominant site of non-shivering thermogenesis, in which lipids are utilized to generate heat [41]. The chemical generation of heat, independent from muscle contraction, is enabled by the BAT-specific factor Uncoupling Protein 1 (UCP1), located in the inner mitochondrial membrane. UCP1 promotes uncoupling of the oxidative phosphorylation from ATP synthesis, which results in leaked protons re-entering the mitochondrial matrix to generate heat instead of chemical energy [42].

Beige adipocytes are characterized as a metabolically intermediate state between white and brown adipocytes and have been under intense investigation over the past decade due to their potentially beneficial role in energy metabolism. These cells do not form a distinct fat depot but rather arise within the WAT, where they differentiate from white adipocytes into beige or brite adipocytes, termed “browning” [43]. In rodents, most beige adipocytes are found in the scWAT; however, in humans, it seems that browning primarily occurs in the visWAT [44]. It remains to be elucidated if beige adipocytes arise de novo from resident precursor cells or if they transdifferentiate from preexisting mature white adipocytes. However, studies put forward evidence for both origins [45,46,47,48]. BAT activation and WAT browning can be induced by different stimuli, such as exercise, chronic cold exposure, dietary regimen, or pharmacological interventions [49,50].

## 3. The Potential of BAT Activation and WAT Browning to Improve Insulin Sensitivity

The processes of BAT activation and WAT browning bear the potential to reestablish metabolic flexibility in diabetic and obese individuals. Most mechanistic studies, in particular those describing cold exposure-mediated stimulation of BAT activity and/or WAT browning, have been conducted in rodents. This chapter aims to summarize the mechanistic background of BAT activation and WAT browning in response to different external stimuli and to assess their specific potential to improve IR or obesity.

## 4. Cold-Induced BAT Activation and WAT Browning

Rodent studies demonstrate increased vascularization in both BAT and WAT in response to low temperatures. Furthermore, cold exposure results in elevated mitochondrial density and reduced fat cell size in white adipocytes, indicating enhanced WAT browning, leading to an increase in energy expenditure, and may therefore potentially ameliorate insulin sensitivity [51]. Of note, beige adipocytes have been shown to retain an “epigenomic memory”. Once they undergo the temperature-dependent reprogramming from white to brown-like adipocytes, they can quickly initiate their thermogenic potential when re-exposed to the cold [52]. Several molecular pathways are being discussed linking the environmental temperature to the metabolic state of the diverse adipose tissues [53,54].

### 4.1. β-Adrenergic Receptor Stimulation

In response to a cold environment, neuronal signals are transduced to the thermoregulatory center located in the hypothalamus, thus activating the sympathetic nervous system (SNS) [55]. The SNS innervates the adipose tissue and releases the catecholamine hormone norepinephrine [56]. Norepinephrine stimulates β3-adrenergic receptors (AR) in adipocytes, leading to increased cyclic AMP (cAMP), which activates the cAMP-dependent protein kinase PKA. PKA mediates phosphorylation of lipolytic enzymes, therefore increasing availability of the substrates for thermogenesis and activation of UCP1 [57,58]. β3-AR signaling is a well-established concept discovered in rodents to activate thermogenesis [59,60]. Interestingly, a recent study conducted in a human BAT cell model presented the β1-subtype of β-AR as the predominant form rather than β3-ARs, indicating that *UCP1* expression is mainly induced via β1-AR signaling in this cell type [61]. Moreover, another study showed that lipolysis and thermogenesis is mediated via β2-AR signaling in human BAT [60], suggesting a possible species-dependent relevance of the different β-AR isoforms in *UCP1* activation.

### 4.2. Thyroidal BAT Activation and WAT Browning

In addition to the neuronal pathway, cold-induced BAT activity is also stimulated by the release of the thyroid hormones triiodothyronine (T3) and thyroxine (T4) from the thyroid gland. In BAT and WAT, conversion of T4 to the active T3 form is induced by type 2 iodothyronine deiodinase (DIO2) [62,63,64], which is associated with a thermogenic response in BAT and WAT [65]. Interestingly, thyroid-mediated WAT browning seems to be independent of *UCP1* as well as SNS-activation and might rather be explained by actions in skeletal muscle via a yet unknown mechanism [66]. However, thyroid hormones have been shown to activate lipolysis via the PKA pathway [64,67], which results in the release of FAs, which are utilized as energy substrates for non-shivering thermogenesis [57,58].

### 4.3. Adipokine-Mediated BAT Activation and WAT Browning

Cold exposure induces the secretion of several adipokines. Among those, fibroblast growth factor 21 (FGF21), which is mainly secreted by the liver [68], has also been shown to be released from BAT and WAT, stimulating expression of thermogenic genes in rodent BAT such as *Ucp1*, *Cidea*, and *Ppargc1a* [69,70]. However, the exact mechanism behind cold-induced FGF21 secretion remains to be elucidated, since existing data are controversial. A number of studies demonstrated increased FGF21 concentrations after cold exposure [71,72], while others failed to show this effect [73,74]. Noteworthy, FGF21 deficiency in mice has been associated with an impaired adaptation to cold temperatures and reduced WAT browning [70]. Studies revealed that cold-induced browning of WAT is also mediated by Interleukin (IL)-6 via β3-AR signaling in rodents [75], or neuregulin 4 (Nrg4) [76,77]. Adiponectin is another adipokine associated with cold-induced browning of scWAT by promoting M2 macrophage proliferation that enhances the production of catecholamine in rodents [78]. Moreover, the expression of bone morphogenetic protein 8b (BMP8b), which is secreted from BAT [79], promotes norepinephrine signaling and induces the secretion of orexin in the hypothalamus stimulating BAT thermogenesis [80].

### 4.4. Cold-Induced Gene Expression in BAT and WAT

In rodents (Table 1), cold-induced BAT activation results in significantly higher expression levels of genes regulating lipid metabolism and thermogenesis, such as *Ucp1*, *Ppargc1a*, *Elovl3*, *Dio2*, *Cebpd*, and *Ppara* in BAT [81,82,83]. The same studies however, failed to show an increased gene expression of *Cidea* and *Prdm16,* which are crucial factors in the induction of the thermogenic program in adipose tissue [82,84,85]. Interestingly, in response to cold exposure, rodent beige adipocytes in WAT exhibit significantly higher expression levels of beige adipocyte-specific markers, such as *Cd137*, *Tmem26*, *Tbx1*, and *Epsti1* compared to BAT, further demonstrating the cold-induced browning process [47,86]. In humans, cold exposure has been shown to induce elevated expression levels of *UCP1* and *TMEM26* in scWAT, promoting a beige adipocyte phenotype [86]. In comparison to scWAT, various research groups have described the gene expression profile of activated human BAT, demonstrating high expression levels of *UCP1*, and transcription factors *ZIC1* and *LHX8* [86]. Most studies demonstrated activated human BAT in response to cold exposure, which is mostly assessed by deoxy-2-[18F]fluoro-d-glucose ([18F]FDG)-positron emission tomography/computed tomography (PET/CT) imaging [87]. However, only few studies show alterations in human WAT in response to cold exposure (Table 2).

### 4.5. Cold Exposure as Potential Treatment of Metabolic Diseases

A large number of rodent studies provide evidence for cold-induced improvements in whole-body glycaemia via enhancement of thermogenic activity in WAT or BAT. In mice, deletion of the transcriptional suppressor of the adipocyte thermogenic program, *zinc finger protein 423 (Zfp423)*, results in visWAT browning. In this study, browning of this WAT depot improved cold tolerance as well as IR in diet-induced obesity. These findings indicate a novel strategy to combat IR in obesity by engineering thermogenic visceral white adipocyte precursors [88]. In addition to the beneficial impacts in mitochondrial biogenesis and function, cold exposure leads to clearance of serum triacylglycerol (TAG) as well as increased glucose uptake into BAT due to increased expression of GLUT4 [89,90,91]. Importantly, increased glucose uptake into BAT was also seen in obese, glucose-intolerant and hypertriglyceridemic apolipoprotein A5 (*Apoa5)*-deficient mice. In fact, cold-induced glucose uptake into BAT of these animals was even higher than in all other tissues combined [89,91]. These data show that cold exposure represents an efficient BAT activator with the potential to improve insulin sensitivity and reduce adiposity, thus indicating clinical relevance for subjects with T2DM or obesity. However, studies have shown that low ambient temperatures may not necessarily exert beneficial effects on whole-body glycaemia [92,93,94].

## 5. Exercise-Induced BAT Activation and WAT Browning

Aerobic endurance as well as resistance exercise training are well-known tools to improve metabolic health by decreasing adipose tissue mass [95] and increasing muscle mass. Studies indicate greater exercise-mediated benefits on metabolic health with higher energy expenditure induced by higher exercise intensities, e.g., during high-intensity interval training (HIIT) [96] and improving insulin sensitivity in a large number of different tissues [97]. In both rodent and human cohorts, chronic exercise training leads to a decrease in adipocyte size and a reduced lipid content in WAT [95,98,99,100,101]. It has been speculated that, as a consequence, thermal insulation is reduced and UCP1-dependent heat generation in WAT and BAT is upregulated [102]. However, apart from these rather indirect influences, exercise has been shown to also directly activate the SNS and therefore potentially stimulate BAT activity and WAT browning via β3-AR in humans and rodents [103,104]. The segregation of the multitude of beneficial impacts of exercise on diverse organs on the molecular level, however, is complex [105,106]. Thus, tracking a metabolic improvement specifically to enhanced BAT activation or WAT browning following an exercise intervention is hardy possible due to the large number of other pathways that are affected in addition [107]. Nonetheless, there is evidence for a contributory role of WAT browning on exercise-related improvements in whole-body glycaemia, mainly dependent on the differential secretion of specific myokines during or post training [108].

### 5.1. Exerkine-Mediated BAT Activation and WAT Browning

Skeletal muscle-adipocyte crosstalk has been established as a crucial axis of glycemic control. The importance of this interaction is emphasized by the finding that exercise-trained scWAT increases skeletal muscle glucose uptake in rodents and humans [109,110]. Regular physical activity increases mitochondrial biogenesis, oxidative capacity, and the number of beige adipocytes in WAT. This exercise-mediated browning is thought to mainly result from myokines or other secreted factors, which have also been termed “exerkines” when secreted in response to exercise [111]. These include for instance metorin-like 1 [112], myostatin [113], and β-aminoisobutyric acid [114]. The exerkine myonectin has been shown to aggravate the abundance of fatty acid transporters FAT/CD36 and FATP1 in rodent scWAT, resulting in lower levels of circulating free fatty acid (FFA) concentrations [115].

In rodents, the skeletal muscle- and adipocyte-secreted factor irisin has been shown to promote thermogenesis-related gene expression in cultured white adipocytes [116] via mitogen-activated protein kinase (P38 MAPK), which activates transcription factor 2 (ATF2), and extracellular signal-associated kinases (ERK), respectively [117]. Irisin secretion is triggered by the exercise-stimulated gene expression of ATF2-dependent peroxisome proliferator-activated receptor γ (PPARγ) coactivator-1 α (PGC-1α), a key regulator of mitochondrial biogenesis and function, which is regulated by a number of physiological and environmental factors [118]. PGC-1α promotes secretion of fibronectin type III domain containing 5 (FNDC5) in skeletal muscle. FNDC5 is cleaved to subsequently yield mature irisin [116]. In mice, it has been demonstrated that elevated irisin levels after exercise induce *Ucp1* mRNA expression and the abundance of adipocytes expressing *Ucp1* in scWAT. These animals also showed enhanced insulin sensitivity and glucose metabolism, linking improved glucose homeostasis to exercise-induced browning of scWAT [116]. These findings were supported by other studies, which presented that attenuating exercise-induced secretion of irisin by either an anti-FNDC5 antibody or loss-of-function *Fndc5*-mutation leads to impaired WAT browning [117] and impaired exercise-induced metabolic improvements [119]. In contrast, treatment with irisin led to elevated WAT browning and decreased fat cell size in mice. Furthermore, blood glucose and cholesterol levels of mice with diet-induced obesity were decreased after irisin treatment [120]. Although some studies failed to show elevated levels of irisin after endurance exercise training in human subjects [121,122,123,124], most others demonstrate increasing circulating irisin levels in response to exercise in humans, which however seems to be dependent on intensity and duration [125,126,127].

PGC-1α plays an important role in exercise-mediated WAT browning and is a key factor in the regulation of cellular energy metabolism, and more specifically, in mitochondrial biogenesis. Therefore, studies in transgenic PGC-1α mouse models help to understand the mechanistic link between exercise-induced secretion of myokines and adipose tissue browning. In contrast to wild type mice, exercised global PGC-1α knockout mice did not express *Ucp1* in WAT in response to exercise [128], emphasizing the relevance of PGC-1α as a crucial regulator of exercise-induced WAT browning. Moreover, depletion of PGC-1α in mice led to a lack of *Ucp1* expression and other browning factors in WAT [129]. Interestingly, tissue-specific ablation of PGC-1α in WAT has been shown to promote whole-body IR in high-fat diet-fed mice, indicated by elevated circulating lipids, simultaneously impaired FA uptake in WAT, as well as impaired inhibition of hepatic glucose output [130]. These results further acknowledge the essential role of PGC-1α in WAT and the link between exercise-induced WAT browning and improvements of whole-body insulin sensitivity.

Members of the IL family of cytokines have been described to play important roles in exercise-stimulated browning and skeletal muscle-adipose tissue crosstalk. Among those, IL-15 is released in response to contraction in lean and obese individuals [131,132,133] and has been shown to promote insulin-stimulated glucose uptake in C2C12 murine skeletal muscle cells [134]. In rodents, administration of IL-15 led to a 35% reduction in WAT mass and a 20% decline in plasma triglycerides, indicating that IL-15 additionally exerts a regulatory role in lipid metabolism [135]. In rats, IL-15 has also been demonstrated to stimulate *Ucp1* gene expression in BAT [136]. IL-6 presents another exerkine regulating WAT browning [137,138]. In contrast to wild type controls, IL-6 knockout mice did not display exercise-induced increased *Ucp1* mRNA expression levels as well as UCP1 protein content [137]. Interestingly, in humans, IL-6 has not only been demonstrated to be secreted in response to exercise interventions by skeletal muscle but also by the adipose tissue [139]. Blocking of IL-6 receptors in human WAT resulted in decreased expression of browning marker genes such as *UCP1* and *CIDEA*. This indicates that IL-6 holds a regulatory function in WAT browning [140]. Of note, also a number of other adipokines have been described to be secreted upon physical activity. For instance, exercise-induced improvements in glucose homeostasis correlated with the secretion of transforming growth factor-β2 (TGF-β2), an adipokine shown to be released from scWAT. A study presented improved glucose tolerance when sedentary mice were transplanted with scWAT from trained wild type but not from trained TGF-β2 knockout animals. However, the exact role of TGF-β2 in obesity, T2DM, and exercise-mediated adaptations remain to be elucidated [141].

### 5.2. Exercise-Induced Gene Expression in BAT and WAT

Studies regarding the effects of exercise on BAT activation are controversial. No study so far described the impact of exercise on BAT activation in obese or type 2 diabetic human subjects [142]. Some rodent studies show that endurance training stimulates the abundance of proteins involved in insulin signaling in BAT, but these studies lack functional evidence such as glucose uptake measurements [143,144]. Other studies even failed to show an increase in glucose transporter type 1 (GLUT1) or GLUT4 after moderate endurance exercise in BAT [104,145]. On the other hand, it was demonstrated that exercise increases BAT activity and gene expression of the thermogenic and mitochondrial markers *Ucp1*, *Dio2*, *Prdm16*, and *Ppargc1a* in rodent BAT [146]. However, there is no evidence to date for a positive impact of exercise on BAT activation in humans (Table 2). Moreover, contrary to expectations, one study demonstrated that endurance exercise led to decreased BAT activity and insulin-stimulated glucose uptake into human BAT. Simultaneously, no changes were detectable in gene expression levels of the mitochondrial markers *PPARGC1A*, *TMEM26*, *CIDEA*, and *CD137* [147]. In WAT, exercise results in significant changes in a number of genes affecting mitochondrial function, beiging, glucose metabolism, and lipid oxidation. Numerous studies presented an exercise-dependent upregulation of *Ucp1*, *Prdm16*, *Cidea*, *Elovl3*, *Cox8b*, *Dio2*, *Cebpb*, and *Ppargc1a* mRNA expression levels in rodent WAT after acute exercise and endurance training [116,128,145,148,149]. In accordance, there is also evidence for a stimulatory role of moderate endurance training on gene expression levels of the browning markers *UCP1*, *TBX1*, and *CPT1B* in human WAT [150]. A selection of relevant studies demonstrating the impacts of exercise interventions on BAT activation and/or WAT browning in rodents are summarized in Table 1.

## 6. Diet-Induced BAT Activation and WAT Browning

In addition to a cold environment and exercise training, thermogenesis in BAT can also be induced by dietary interventions in both rodents (Table 1) and humans (Table 2). Catechins have been shown to reduce body fat mass by 2–3% in humans when ingested regularly [151,152,153,154]. Moreover, several studies describe specific dietary components such as menthol [155,156,157,158] or capsaicin, found in spicy foods [159,160], whose consumption leads to an activation of UCP1-dependent thermogenesis in human BAT and to the prevention of high-fat diet-induced obesity. However, these studies did not address if thermogenesis and prevention of obesity were causally related to each other. Dietary supplementation with branched-chain amino acids (BCAAs), such as leucine (Leu) and isoleucine (Ile), have been demonstrated to reduce body fat mass and stimulate WAT browning in mice. Furthermore, Leu and Ile supplementation decreased lipid accumulation and increased inulin sensitivity, which was assessed by glucose and insulin tolerance tests. These findings support the idea that dietary BCAAs play a role in the management of obesity and metabolic disorders [161].

However, not only specific dietary components are capable of inducing BAT activation and potentially stimulating WAT browning. Feeding behavior has also been shown to have direct effects on the metabolic activity of adipose tissue. In rodents, leptin, an adipokine secreted in response to insulin, has recently been demonstrated to act on the hypothalamus, stimulate distinct sympathetic nerves in BAT, and, in addition, enhance WAT browning [162]. Furthermore, a recent study demonstrated that mice subjected to an intermittent fasting regimen, with no alteration in cumulative food intake compared to the *ad libitum* control group, showed increased abundance of beige adipocytes within WAT depots. This fasting regimen further reduced body weight and improved IR in lean mice and metabolic syndrome in obese mice [163].

**Table 1:** Impacts of cold exposure, exercise interventions, dietary composition, and pharmacological compounds on BAT and WAT metabolism in rodents.

**Cold Exposure**				
**Duration and Temperature**	**Experimental Animal**	**BAT**	**WAT**	**Reference**
4–5 days (4 °C)	lean C57BL/6 mice	increased angiogenesis	N/A	[164]
1 week (4 °C)	lean C57BL/6 mice	increased angiogenesis	high abundance of UCP1 and mitochondria	[165]
6 days (5 °C)	lean C57BL/6 mice	N/A	positive UCP1 staining	[166]
10 days (6 °C)	lean C57BL/6 mice	increased gene expression (*Ucp1, Prdm16, Ppargc1a, Cidea, Pparg2, Dio2*)	positive UCP1 staining	[167]
10 days (6 °C)	129Sv mice	increased gene expression (*Ucp1, Prdm16, Pgc1a, Cox8b*)	positive UCP1 staining	[168]
4 wks (5 °C)	rats	Increased activity	N/A	[169]
**Exercise Training**				
**Training Protocol**	**Experimental Animal**	**BAT**	**WAT**	**Reference**
5 day/wk for 8 wks (moderate endurance training)	C57BL/6 mice (HFD, ND)	increased *Ucp1* expression	increased gene expression (*Ucp1, Prdm16, Ppargc1a, C/ebpβ, Pparg2, Dio2*)	[170]
5 day/wk for 8 wks (aerobic or resistance exercise training)	Swiss mice	decreased weight + lipid area	increased angiogenesis, UCP1, CD31 abundance, increased browning marker gene expression	[171]
2 h daily swim for 4 wks	Wistar rats	N/A	*Ppargc1a* and *Tfam* mRNA expression	[172]
4-weeks of swim-training (1 h/day, 5 days per week)	Sprague-Dawley rats (HFD)	no effect	no effect	[173]
3 wks (voluntary running wheel)	mice	decreased mitochondrial activity and GU	increased UCP1 abundance, mitochondrial/beige gene expression (scWAT)	[145]
**Diet**				
**Diet Composition**	**Experimental Animal**	**BAT**	**WAT**	**Reference**
high-fat diet (n-3 PUFA)	rats	increased thermogenic activity	N/A	[174]
single dose (capsiate or capsaicin)	Wistar rats	increased sympathetic nerve activity (SNA), increases *Ucp1* mRNA expression	N/A	[159]
dietary resveratrol (10 wks)	db/db mice	increased UCP1 abundance	increased UCP1 abundance	[175]
diet supplemented with 0.4% resveratrol	obese mice	N/A	increased UCP1 abundance	[176]
**Pharmacological Compounds**				
**Compound**	**Experimental Animal**	**BAT**	**WAT**	**Reference**
β1-adrenoceptor agonist xamoterol hemifumarat	mixed 129Sv × C57BL/6 mice	N/A	N/A	[168]
β3-adrenoceptor agonist CL316,243		N/A	increased UCP1 abundance	
CL316,243 (7 days)	Sprague-Dawley rats	increased UCP1 abundance	increased UCP1 abundance	[177]
CL316,243 (6 days)	C57Bl/6 mice and 129S1/SvImJ	minimally increased UCP1 abundance	increased mitochondrial biogenesis + abundance of multilocular adipocytes	[178]
4-methylumbelliferone (4-MU)	C57Bl6/J mice	Increased activity	increased UCP1 abundance, increased *Ucp1*, *Ppargc1a*, *Tbx1*, and *Tmem26* gene expression	[179]

### WAT Browning as Potential Opponent of Age-Dependent Insulin Resistance

Ageing represents an important risk factor for the development of a variety of metabolic diseases. IR has been associated with increasing senescence of adipocytes and age [180,181]. Ageing leads to a decrease in BAT tissue mass and activity, eventually resulting in impaired and deregulated glucose and lipid metabolism [89,182,183,184]. In mice, WAT browning has been demonstrated to be disabled due to aging [185]. The ageing-related reduced WAT browning in mice and humans has been suggested to be caused by lacking *CD137/TMEM2* expressing progenitor cells, which display a subpopulation of white progenitors that are more likely to differentiate into beige adipocytes [186]. In humans, it was further shown that expression levels of other genes regulating WAT browning decrease during ageing, including *SIRT1* and *PRDM16* [187]. Therefore, therapeutical BAT activation and WAT browning may contribute to preventing age-related IR in the future.

## 7. Therapeutical Potential of BAT Activation and WAT Browning in Humans

Due to the severe health impairments related to obesity, a multiplicity of therapeutical approaches to target obesity and its comorbidities have been developed over the years. Among those, pharmacological interventions to reduce appetite and thus lower body weight, especially body fat mass, have been valiant efforts. However, many of these medications are associated with unwanted side effects on the neuronal or cardiovascular system, including depression or heart diseases [188]. Therefore, identifying non-pharmaceutical anti-obesity and -diabetes approaches to retain metabolic flexibility and a healthy inter-organ crosstalk are crucial in the attempt to improve public health. The stimulation of metabolically active BAT or the increase of brown-like adipocytes offers great potential to increase whole-body energy expenditure and decrease adipose tissue mass by increased lipid utilization. In obese individuals, both increased BAT activation and browning could lead to utilization of excess lipids to generate heat and prevent pathological lipid accumulation. By preventing or potentially counteracting ectopic fat accumulation in insulin-responsive tissues, browning and activated BAT may lead to utilization of harmful lipid metabolites, which would otherwise interfere with the insulin signaling cascade. However, up to today, a direct impact of WAT browning on ectopic lipid accumulation in peripheral tissues or stimulatory effects on the insulin signaling cascade have not been demonstrated. However, there is evidence for promising therapeutical approaches based on BAT activation or WAT browning. Table 2 shows studies conducted in human subjects analyzing the effects discussed in the following section on BAT activity and WAT phenotype. Furthermore, the following section aims to summarize the current status of these potential treatment options.

### 7.1. Therapeutical Approach A: β-Adrenergic Receptor Stimulation by Cold Exposure

In humans, physiological responses to cold exposure clearly depend on the duration of the respective intervention. In response to acute cold exposure, cutaneous vasoconstriction and shivering thermogenesis will result in decreased heat loss and elevated metabolic heat generation. Acute cold exposure was shown to promote increased gene expression levels of *PPARGC1A* and *UCP1* in scWAT biopsies of healthy but not obese individuals [92]. Furthermore, cold-induced BAT activation is associated with improved insulin sensitivity in humans by increasing peripheral glucose uptake, which interestingly seems to be independent of pancreatic insulin secretion in healthy individuals [93,94]. In fact, a number of studies demonstrated BAT activity in response to cold exposure [189,190]. When exposed to the cold, physiological responses have been shown to adapt. Habituation can be a major drawback especially in chronic cold exposure investigations, as responses to cold are less pronounced after adaptation [191]. Chronic cold exposure results in metabolic acclimatization including pronounced thermogenic response, enhanced non-shivering thermogenesis, and increased metabolic rate in adipose tissue. These factors have been shown to be dependent on the duration of cold exposure and influenced by human sex, race, fitness, and thermoregulatory fatigue [191].

Few studies have been investigating the impacts of prolonged cold exposure on WAT browning in human subjects. One study conducted in healthy humans showed that cold exposure (15 °C–16 °C, 2 h to 6 h/day, 10 days) failed to induce browning in scWAT [192]. Another study demonstrated that after 11 days of daily cold exposure (2 h per day) norepinephrine response was reduced by 20% [193]. In contrast to that, daily exposure of subjects for 2 h over 10 days to temperatures 1–2 °C above the onset of shivering, which is usually between 16 and 19 °C, resulted in activated BAT but no increase in UCP1 expression in scWAT was observed [87]. Additional cooling vests can be used for this “fixed type cooling protocol” to stimulate BAT activity [87,192,194,195]. For obese individuals, due to their extra subcutaneous fat, this protocol might not be sufficient. Therefore, a water cooling approach was suggested to be more promising [196]. Interestingly, the “fixed type cooling protocol” has been shown to improve skeletal muscle insulin sensitivity by 43% in T2DM patients. However, this result was not achieved due to BAT activation but rather increased GLUT4 translocation [197]. Interestingly, when shivering was prevented in T2DM patients, these beneficial effects on skeletal muscle insulin sensitivity were ablated [198]. This indicates that BAT activation is associated with muscle action, which further suggests that exerkines affect the phenotype of WAT including browning [199]. These findings further indicate the difficulty in identifying browning-specific improvements in glucose or lipid metabolism. Another study demonstrated that daily 2 h cold exposure at 17 °C for 6 weeks increased BAT activity and further led to increased energy expenditure and decreased body fat mass in healthy humans [200].

While it seems that long-term cold exposure may promote beiging of scWAT in humans, this treatment approach might take more time to achieve the desired effects. Therefore, patients may not tolerate this approach as well as an exercise intervention. Furthermore, cold-induced vasoconstriction increases cardiovascular strain and decreases myocardial oxygen supply in individuals with coronary artery disease. While effects of cold-expose on subjects with cardiovascular diseases are not well known, cold interventions might not represent a suitable strategy to induce browning in these subjects [201]. However, further studies are needed in order to define the specific duration of cold exposure that is sufficient to induce beiging in humans and whether these observations can be translated to subjects with obesity and T2DM.

### 7.2. Therapeutical Approach B: β-Adrenergic Receptor Stimulation by Pharmacological Compounds

Findings from cold exposure studies indicate that both activation of brown adipocytes and an increased number of beige adipocytes in WAT play a role in thermogenesis, regulation of lipid metabolism, and whole-body glycaemia in humans [192]. From a mechanistic point of view, stimulation of β-AR represents the most important link between cold exposure and enhanced mitochondrial activity in WAT or BAT. Activation of β1- and 2-AR has been shown to regulate lipolysis and thermogenesis in humans by increasing UCP1-dependent oxidative metabolism and glucose uptake into BAT. In contrast to rodents, where stimulation of the β3-AR subtype seems to be the predominant pathway to stimulate BAT activation and WAT browning, β1- and 2-AR subtypes have been described as more relevant for non-shivering thermogenesis in humans [60,61]. However, clinical evidence gained in pharmacological studies indicate a potential relevance of β3-AR activation in humans as well (Table 2). For instance, the selective β3-AR agonist Mirabegron (Myrbetriq) has been demonstrated to stimulate BAT activation in a cohort of lean and obese individuals. Moreover, this substance was shown to increase in vivo energy expenditure, plasma FFA concentration, and glucose uptake into BAT of these subjects [202,203,204,205]. In obese individuals, Mirabegron treatment increased *UCP1* gene expression levels in scWAT [203] and chronic Mirabegron treatment (100 mg daily/4 weeks) resulted in improved insulin sensitivity, glucose tolerance, and insulin secretion measured during an intravenous glucose tolerance test [205]. It has to be noted that Mirabegron is a medication for urinary urgency or incontinence and potentially has unwanted side effects such as increased heart rate that have not been fully evaluated yet [202]. Consequently, in addition to Mirabegron as a β3-AR-agonist, application of β1- or 2-AR-agonists might also have clinical relevance for the treatment of metabolic disorders in human. However, so far, no data from human cohorts are available.

### 7.3. Therapeutical Approach C: Exercise

Physical activity holds multiple beneficial effects on overall metabolic health. In response to muscle contraction, approximately 3000 myokines are differentially regulated [206]. However, only a small fraction of the wide range of interconnecting pathways affected by myokines have been elucidated up to today. Some attempts, however, were successful in showing a beneficial relationship between exercise, myokine secretion, and WAT browning (Table 2). The most famous example for a circulating factor affecting WAT browning and thus improving metabolic health is the PGC-1α-dependent myokine irisin. Irisin has been shown to be secreted from exercising skeletal muscle in a number of rodent studies [207]. Moreover, it has also been shown to be released from murine adipose tissue [208]. In humans, gene expression of FNDC5/irisin is 100–200 times higher in skeletal muscle compared to WAT [116,209]. However, studies demonstrated that obese individuals show elevated circulating irisin levels, which most likely result from the enlarged fat depots that may contribute to increased irisin levels [208]. These chronically elevated irisin levels in the obese state can lead to adipose tissue and skeletal muscle irisin resistance. Therefore, obese individuals may not benefit from irisin-mediated metabolic improvements, including increased expression of browning-related genes in mature adipocytes and increased energy expenditure, to the same extent as lean persons [210].

IL-6, another myokine that has been under intense investigation during the last decade, holds both functions as a pro-inflammatory cytokine and an anti-inflammatory factor [211]. Treatment with IL-6 leads to increased insulin-stimulated glucose uptake and FAO in skeletal muscle of healthy humans [212,213]. Moreover, the same study showed that incubation with IL-6 results in enhanced basal and insulin-stimulated glucose uptake due to increased GLUT4 translocation and increased AMP-activated protein kinase (AMPK) activity in L6 myotubes [213]. However, the role of IL-6 on glucose metabolism are controversial and the large number of studies differing in the respective model, protocol, and condition impede a clear-cut interpretation. For instance, several studies indicate that acute IL-6 treatment fails to improve insulin-stimulated glucose uptake in the skeletal muscle of healthy humans [212,214].

There are a number of in vitro studies, which describe impacts of diverse exercise-induced myokines on glucose homeostasis. Myokine-based approaches seem to be promising as a therapeutic tool to improve overall metabolic health by promoting beige adipocyte differentiation [140]. However, a lot of work remains to be done to define myokines, their role in regulating glucose homeostasis, and elucidate their potential in re-establishing a healthy inter-organ crosstalk during T2DM or obesity.

**Table 2:** Impacts of cold exposure, exercise interventions, dietary composition, and pharmacological compounds on BAT and WAT metabolism in humans.

**Cold Exposure**				
**Duration and Temperature**	**Health Status**	**BAT**	**WAT**	**Reference**
10 days (16–17 °C)	T2DM	no activity	N/A	[198]
10 days (14–15 °C)	T2DM	minor activity; increased GU	N/A	[197]
5–8 h	healthy	activated (increased GU)	N/A	[189]
1 month (19 °C–10 h/day)	healthy	activated (PET/CT)	N/A	[215]
acute cold exposure (time 120–300 min)	healthy, T2DM	increased oxidative metabolism and radiodensity	N/A	[216]
4 wks (10 °C 2 h daily–5 d/wk)	healthy	increased GU, 45% increase in volume of activity	N/A	[217]
6 wks (17 °C 2 h/day)	healthy	Increased activity	N/A	[218]
2 h	Athletes vs. sedentary	Lower in athletes	No changes	[147]
10 days (15–16°C 6 h/day)	healthy	Increased activity	No changes (UCP1)	[192]
10 days (30 min/day–local application)	lean and obese	N/A	Increased UCP1 and TMEM26 abundance	[203]
**Exercise Training**				
**Training Protocol**	**Health Status**	**BAT**	**WAT**	**Reference**
3 days/wk for 12 wks (bicycle, intensity 70–80% HRmax)	non-diabetic (normal weight to obese)	N/A	increased gene expression (*UCP1, TBX1, CPT1B*), positive P2rx5 staining	[150]
6 sessions over 2 wks (high-intensity interval training (HIIT) and moderate-intensity continuous training (MICT)	healthy	decreased insulin-stimulated GU	N/A	[219]
**Diet**				
**Diet Composition**	**Health Status**	**BAT**	**WAT**	**Reference**
capsinoids (6 wks daily)	healthy	N/A	N/A	[218]
capsinoid (12 wks–6 mg/d)	mildly obese	N/A	N/A	[220]
**Pharmacological Compounds**				
**Compound**	**Health Status**	**BAT**	**WAT**	**Reference**
Mirabegron (Myrbetriq)	healthy	Increased activity	Increased adiponectin secretion	[205]
Mirabegron (Myrbetriq)	lean and obese	N/A	Increased UCP1, TMEM26, and CIDEA abundance	[203]

## 8. Conclusions—Let’s Get Real about WAT Browning as Therapeutic Approach

In the past, many reviews have highlighted the relevance of WAT browning and BAT activation on metabolic health. Especially in rodents, the correlation of cold- and exercise-induced browning and improved glucose as well as lipid metabolism was largely discussed. However, the focus was usually set on the effects of a specific browning stimulus and not on a comparative overview of different stimuli. Moreover, often translational aspects were missing. In this review, we provide an overview on the pathways of cold-, exercise-, and diet-induced stimulation of BAT activation, WAT browning, and their relevance in the prevention and treatment of metabolic diseases in humans. Various studies in rodents associate BAT activity and therefore WAT browning with leanness [221] and normoglycaemia [222,223,224]. It has been observed that activated BAT and WAT browning is associated with increased mitochondrial activity, enhanced energy expenditure, as well as improved clearance of glucose and lipids from the circulation [51,89,90,91,116]. In humans, however, the data presented on WAT browning and its impacts on glucose homeostasis are more controversial and less intensively investigated. Of note, human BAT more closely represents murine beige adipose tissue than classical murine BAT [225,226]. Nevertheless, it has been demonstrated that BAT activation is associated with improved insulin sensitivity in humans by increasing peripheral glucose uptake in healthy [93,94] and T2DM [197] subjects.

Beige adipose tissue and BAT can be activated by a range of different stimuli. In rodents, cold exposure has been shown to be the most potent inducer of non-shivering thermogenesis to generate heat via mitochondrial UCP1 in BAT and WAT [142]. In contrast to rodents, human data on cold-mediated WAT browning are not consistent [192,200], which may be due to fact that in rodent studies the experimental animals are subjected to extremely low temperatures (approximately 4 °C) in comparison to their thermoneutrality (30 °C), while humans are subjected to temperatures of 16–19 °C when shivering is prevented.

Regular exercise has been well described to recruit beige cells and to be associated with improved glycaemia in rodents and humans. These finding indicate promising therapeutic strategies to improve glycaemia in human subjects due to a minimum of unwanted side effects compared to most medications. The advantage of WAT browning, compared to classical BAT activation, lies in the high abundance of WAT in the human body that has the potential to brown, resulting in an enhanced level of energy expenditure. In addition, browning has been demonstrated to be independent of insulin signaling; therefore, proving the therapeutic potential of increasing energy expenditure and improving glucose homeostasis during insulin resistance. For obese T2DM subjects, a combination of chronic cold exposure and exercise training could be the most effective way to improve overall metabolic health. Exercise will reduce the scWAT insulator, thus the cold intervention can effectively enhance shivering and non-shivering thermogenesis. However, little is known about browning-specific improvements in whole-body glucose and lipid metabolism as well as the longevity of the positive effects of WAT browning so far. Thus, further investigations on the physiological and metabolic relevance of the interconnecting pathways between BAT activity, WAT browning, and glycaemia are needed to show clinical relevance in the treatment of obesity and T2DM.
